# Enhanced Hypercoagulability Using Clot Waveform Analysis in Patients with Acute Myocardial Infarction and Acute Cerebral Infarction

**DOI:** 10.3390/jcm13237181

**Published:** 2024-11-26

**Authors:** Jun Masuda, Hideo Wada, Takashi Kato, Yusuke Tanigaito, Koken Hayashi, Keita Yamada, Keigo Nishida, Hiroki Oizumi, Toshitaka Kamon, Takanobu Ohkubo, Karin Okamoto, Nobuo Ito, Katsuya Shiraki, Yuhuko Ichikawa, Motomu Shimaoka, Kaoru Dohi, Hideto Shimpo

**Affiliations:** 1 Department of Cardiology, Mie Prefectural General Medical Center, Yokkaichi 510-8561, Japan; jun-masuda@mie-gmc.jp (J.M.); takeshi-kato@mie-gmc.jp (T.K.); yusuke-tanigaito@mie-gmc.jp (Y.T.); koken-hayashi@mie-gmc.jp (K.H.); keita-yamada@mie-gmc.jp (K.Y.); keigo-nishida@mie-gmc.jp (K.N.); hiroki-oizumi@mie-gmc.jp (H.O.); 2 Department of General and Laboratory Medicine, Mie Prefectural General Medical Center, Yokkaichi 510-8561, Japan; katsuya-shiraki@mie-gmc.jp; 3 Department of Neurology, Mie Prefectural General Medical Center, Mie, Yokkaichi 510-8561, Japan; toshi-kamon@mie-gmc.jp (T.K.); takanobu-ohkubo@mie-gmc.jp (T.O.); karin-okamoto128@mie-gmc.jp (K.O.); nobuo-itou@mie-gmc.jp (N.I.); 4 Department of Central Laboratory, Mie Prefectural General Medical Center, Yokkaichi 510-8561, Japan; yuhuko-ichikawa@mie-gmc.jp; 5 Department of Molecular Pathobiology and Cell Adhesion Biology, Mie University Graduate School of Medicine, Tsu 514-8507, Japan; motomushimaoka@gmail.com; 6 Department of Cardiology and Nephrology, Mie University Graduate School of Medicine, Tsu 514-8507, Japan; dohik@clin.medic.mie-u.ac.jp; 7 Mie Prefectural General Medical Center, Yokkaichi 510-8561, Japan; hideto-shimpo@mie-gmc.jp

**Keywords:** CWA, APTT, sTF/FIXa, acute myocardial infarction, acute cerebral infarction

## Abstract

**Background**: Routine activated partial thromboplastin time (APTT) and prothrombin time (PT) measurements do not indicate hypercoagulability in patients with acute myocardial infarction (AMI) and acute cerebral infarction (ACI). **Methods**: Hypercoagulability in patients with AMI or ACI was evaluated using a clot waveform analysis of the APTT or a small amount of tissue factor activation assay (sTF/FIXa). In the CWA, the derivative peak time (DPT), height (DPH), width (DPW), and area the under the curve (AUC) were evaluated. **Results**: The APTT did not indicate hypercoagulability, but the second DPT of CWA-sTF/FIXa was significantly shorter in patients with ACI than in healthy volunteers (HVs). The first DPH values of CWA-APTT and CWA-sTF/FIXa in patients with ACI and AMI were significantly higher than in HVs. In the receiver operating characteristic (ROC) analyses of ACI or AMI vs. non-thrombosis, the AUC was >0.800 in the DPHs of CWA-APTT and CWA-sTF/FIXa. The AUC of CWA-APTT and CWA-sTF/FIXa in patients with AMI and ACI was significantly higher than in HVs. The AUC/second DPT of CWA-APTT and CWA-sTF/FIXa in patients with AMI and ACI was significantly higher than in HVs. Regarding the ROC analyses of ACI or AMI vs. HVs, the AUC of ROC was higher than 0.800 in the AUC and AUC/second DPT of CWA-APTT and CWA-sTF/FIXa. **Conclusions**: The AUC/second DPT of CWA-APTT and CWA-sTF/FIXa may be a useful parameter for detecting a hypercoagulable state in patients with AMI and ACI.

## 1. Introduction

Acute coronary syndrome (ACS) and acute myocardial infarction (AMI) are caused by atherothrombotic coronary artery disease resulting from atherosclerotic plaque rupture or erosion with a non-occlusive/occlusive thrombus [1,2]. AMI, including ST-elevation myocardial infarction (STEMI) [3,4], is generally diagnosed based on clinical symptoms, electrocardiography, biomarkers such as troponin [5] and creatine phosphokinase-MB (CKMB) [6], and coronary angiography [7]. After percutaneous coronary intervention [7], patients with AMI are usually treated with antiplatelet therapy [8,9] and are not considered hypercoagulable, except for those with cancer-related thrombosis (CAT) [10].

Acute cerebral infarction (ACI) [11,12] also includes atherosclerotic ACI [13], which is due to arterial thrombosis; this differs from cardioembolic ACI, which is due to venous thrombosis [14]. Although ACI is usually diagnosed using computed tomography, magnetic resonance imaging, and angiography in core hospitals, the differential diagnosis between cardioembolic ACI and atherosclerotic or lacunar ACI may be difficult [15]. In Japan, patients with atherosclerotic ACI are usually treated with antiplatelet agents [11,16] after treatment with argatroban [17]. Therefore, platelet activation may play an important role in the development of atherosclerosis, resulting in ACI or AMI [18]. In contrast, patients with cardioembolic ACI are treated with warfarin or DOACs [19,20] for a hypercoagulable state [21].

Regarding biomarkers, troponin T [22] and CKMB [6] are sensitive to AMI; however, these biomarkers are for myocardial injury and not for arterial thrombosis. Biomarkers for thrombosis may be more sensitive for myocardial injuries, suggesting that thrombotic biomarkers can detect the early phase of AMI. Elevated D-dimer levels [23,24,25] suggest venous thromboembolism (VTE) and disseminated intravascular coagulation (DIC). Biomarkers for platelet activation may be useful for the diagnosis of arterial thrombosis (e.g., AMI and ACI). Therefore, there have been reports on soluble C-type lectin receptor-2 [15], platelet factor 4 [26], β-thromboglobulin [27], and P-selectin [28]. Furthermore, the detection of hypercoagulability by a clot waveform analysis (CWA)-activated partial thromboplastin time (CWA-APTT) and CWA-small amount of tissue factor (TF) induced FIX activation assay (CWA-sTF/FIXa) has been recently reported in atherosclerotic ACI [21].

In this study, hypercoagulability in patients with AMI and ACI was examined using a CWA, and its usefulness in the diagnosis of AMI and ACI was analyzed by a receiver operating characteristic (ROC) analysis.

## 2. Materials and Methods

Patients with hemostatic abnormalities who were admitted to Mie Prefectural General Medical Center from September 1, 2020 to April 30, 2024 were investigated. Patients with AMI [*n* = 123; mean age ± standard deviation (SD), 68.4 ± 13.7 years; female, *n* = 26; male, n = 97], ACI [*n* = 104; mean age, 75.6 ± 5.1 years; female, *n* = 29; male, *n* = 75], chronic liver disease (CLD, *n* = 148; mean age, 75.6 ± 5.1 years; female, *n* = 73; male, 75), and cancer (*n* = 162; mean age, 74.4 ± 12.1 years; female, 35; male, 127) were examined using a CWA. CWA examinations were also performed on 50 healthy volunteers (HVs; mean age, 45.1 ± 18.0 years; female, *n* = 33; male, 17). Blood samples were obtained during days 1 and 14 post-admission. Patients who were treated with anticoagulants were excluded from the study. Patients with ACI and AMI were treated with antiplatelet therapy. ACI was diagnosed using clinical symptoms, physical examinations, medical history, and computed tomography or magnetic resonance imaging findings. AMI was diagnosed based on clinical symptoms, physical examinations, medical history, electrocardiograms, biomarkers such as troponin T and creatine phosphokinase-MB, and coronary angiography. The study protocol (O-0051) was approved by the Human Ethics Review Committee of the Mie Prefectural General Medical Center, and informed consent was obtained from each participant. This study was conducted in accordance with the principles of the Declaration of Helsinki.

Platelet-rich plasma (PRP) and platelet-poor plasma (PPP) were prepared via centrifugation at 900 rpm and 3000 rpm, respectively, for 15 min [29]. The APTT using PPP was measured using an APTT-SP^®^ (Instrumentation Laboratory, Bedford, MA, USA) with an ACL-TOP^®^ (Instrumentation Laboratory), as previously reported [30,31]. The sTF/FIXa assay using PRP was performed using 2000-fold diluted HemosIL RecombiPlasTin 2G (TF concentration < 0.1 pg/mL; Werfen. A CWA was performed as follows: Three curves (navy, pink, and light blue lines) are displayed on the monitor of an ACL-TOP^®^ system [30,31]. The fibrin formation (FF) curve (navy line) corresponds to the changes in the absorbance observed while measuring the APTT. The first derivative peak (first DP) curve (pink line) corresponds to the coagulation velocity. The second derivative peak (second DP) curve (light blue line) corresponds to coagulation acceleration. The height and time of the FF, first DP, and second DP curves are abbreviated as FFH and FFT, first DPH and DPT, and second DPH and DPT, respectively.

**Figure 1:**
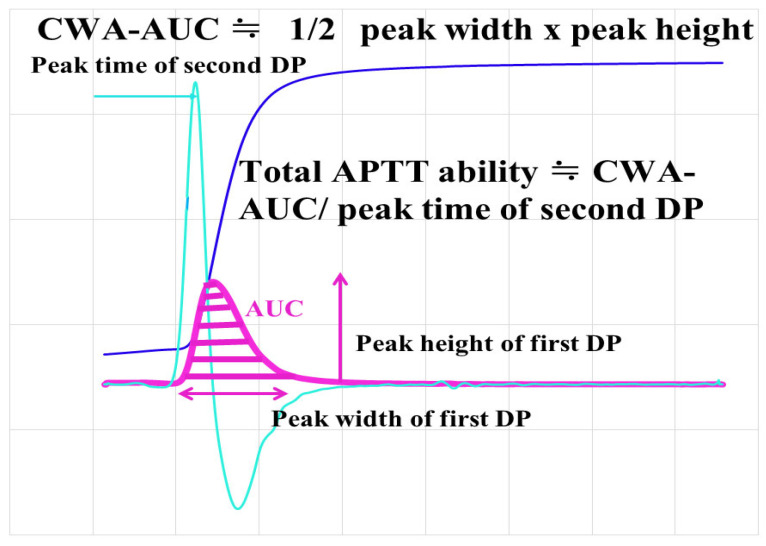
CWA-AUC and total ability of CWA-APTT. APTT, activated partial thromboplastin time; navy line, fibrin formation curve; pink line, first derivative curve (velocity); light blue, second derivative curve (acceleration); AUC, area under the curve.

The area under the curve of the CWA (AUC-CWA) of the first DP indicates the ability of coagulation velocity instead of the peak time or height (Figure 1), and “half of the peak height × peak width” may be similar to AUC-CWA. In addition, “AUC-CWA/second peak time” may show the total coagulation ability, as shown by the APTT. The reference interval was within the 95% confidence interval of the healthy volunteers.

### Statistical Analyses

Data are expressed as the median (range). The significance of differences between groups was examined using the Mann–Whitney *U* test. Cutoff values, determined as the point at which the sensitivity and specificity curves intersected, were examined using a ROC analysis. *p*-values of <0.05 were considered to indicate statistical significance. All statistical analyses were performed using the Stat-Flex software program (version 6; Artec Co., Ltd., Osaka, Japan).

## 3. Results

**Figure 2:**
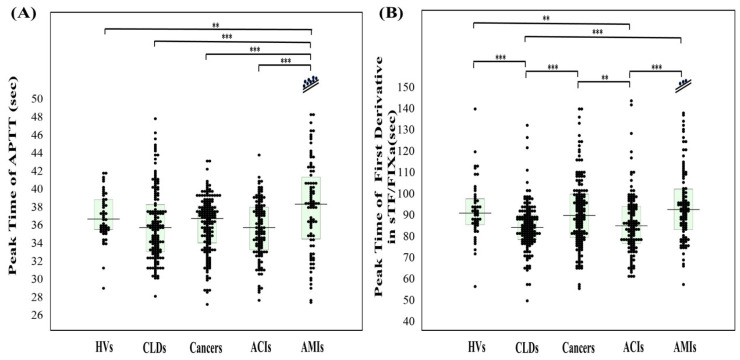
Peak times of APTT (**A**) and sTF/FIXa (**B**) in HVs, and patients with CLD, cancer, ACI, and AMI. APTT, activated partial thromboplastin time; sTF/FIXa, small amount of tissue factor-induced FIX activation assay; HVs, healthy volunteers; CLD, chronic liver disease; ACI, acute cerebral infarction; AMI, acute myocardial infarction; **, *p* < 0.01; ***, *p* < 0.001. Navy line, fibrin formation curve; FF, fibrin formation; pink line, first derivative curve (velocity); first DPH, first derivative peak height; light blue, second derivative curve (acceleration); second DPH, second derivative peak height; solid line, patient; dotted line, control.

**Figure 3:**
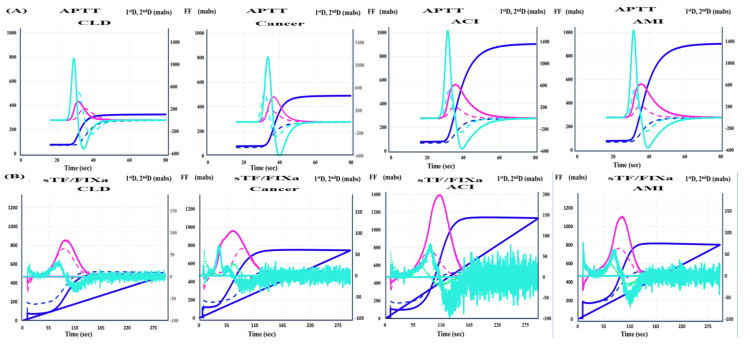
CWA-APTT (**A**) and CWA-sTF/FIXa (**B**) in patients with CLD, cancer, ACI, and AMI. Navy line, fibrin formation curve; FF, fibrin formation; pink line, first-derivative curve (velocity); first DPH, first derivative peak height; light blue, second derivative curve (acceleration); second DPH, second-derivative peak height; solid line, patient; dotted line, control.

The reference interval of APTT (the second DPT of CWA-APTT) has been established as 27.3–35.8 s from 120 HVs, but the establishment of a reference interval for other parameters of CWA-APTT and CWA-sTF/FIXa is still proceeding. The 95% CI of CWA-APTT and CWA-sTF/FIXa in this study were as follows; for the second DPT, 30.7–41.9 s and 48.9–97.7 s, respectively, for the first DPT, 29.0–39.9 s and 68.1–125 s, respectively, for the fibrin formation time, 26.4–49.4 s and 74.9–124 s, respectively, for the second DPH, 434–1007 mabs and 20.4–81.1 mabs, respectively, for the first DPH, 137–317 mabs and 39.5–97.2 mabs, respectively, and for the FFH, 131–258 mabs and 175–519 mabs, respectively. The APTT was not significantly shorter in the patients with ACI and AMI than in the HVs, the patients with CLD, or the patients with cancer (Figure 2A), while it was significantly longer in the patients with AMI and was significantly longer than that in the patients with CLD, cancer, and ACI. The patients with AMI treated with percutaneous coronary intervention may have been affected by heparin. The peak time of the first derivative in CWA-sTF/FIXa was significantly shorter in the patients with CLD and the patients with ACI than in the HVs (Figure 2B). CWA-APTT showed no significant difference (*p* < 0.01) in peak times among the HVs and the patients with CLD, cancer, ACI, and AMI, and the peak heights in the patients with CLD, cancer, ACI, and AMI were significantly higher than those in the HVs (Figure 3A and Table 1). CWA-sTF/FIXa showed that the peak time of the second derivative in the patients with CLD, ACI, and AMI was significantly shorter than in the HVs and that the peak height of the first derivative in the patients with CLD, cancer, ACI, and AMI was significantly higher than in the HVs (Figure 3B and Table 1). In the ROC analyses of ACI or AMI vs. non-thrombosis, the AUC was >0.800 in the second DPH of CWA-APTT (ACI or AMI vs. HVs), the first DPH of CWA-APTT and CWA-sTF/FIXa (ACI or AMI vs. HVs), and the FFH of CWA-sTF/FIXa (AMI vs. HVs or CLD) (Table 2).

**Figure 4:**
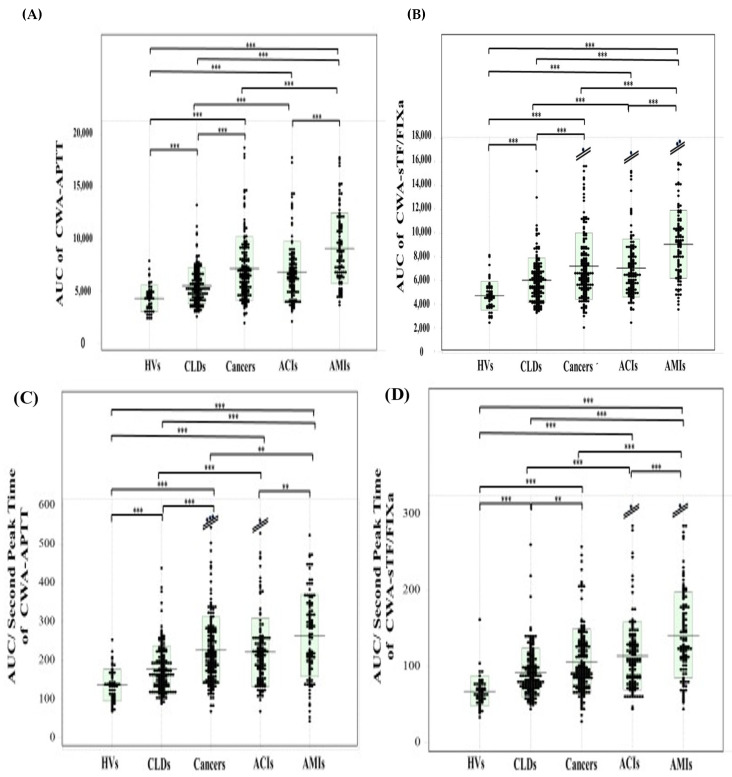
AUC {(**A**,**B**)} AUC/second peak time {(**C**,**D**)} of CWA-APTT {(**A**,**C**)} and CWA-sTF/FIXa {(**B**,**D**)} in HVs and patients with CLD, cancer, ACI, and AMI. APTT, activated partial thromboplastin time; sTF/FIXa, small amount of tissue factor-induced FIX activation assay; HVs, healthy volunteers; CLD, chronic liver disease; ACI, acute cerebral infarction; AMI, acute myocardial infarction; **, *p* < 0.01; ***, *p* < 0.001.

The reference interval of the AUC of CWA-APTT and CWA-sTF/FIXa was 2471–7277 mabs × sec and 2831–8031 mabs × sec, respectively. The AUC of CWA-APTT was significantly higher in the patients with cancer {6592 mabs × sec (5024–8628 mabs × sec)}, AMI {8689 mabs × sec (6518–11,243 mabs × sec)}, and ACI {8419 mabs × sec (4899–7921 mabs × sec)} than in the HVs {4343 mabs × sec (3382–4954 mabs × sec)}and the patients with CLD {5257 mabs × sec (4213–6632 mabs × sec)}, and it was significantly higher in the patients with AMI than in the HVs and the patients with CLD and cancer (Figure 4A). The AUC of CWA-sTF/FIXa was significantly higher in the patients with AMI {9216 mabs × sec (6890–10,748 mabs × sec)} than in the HVs {4643 mabs × sec (3908–5357 mabs × sec)} and the patients with ACI {6530 mabs × sec (5288–8265 mabs × sec)}, cancer {6626 mabs × sec (5377–8601 mabs × sec)}, and CLD {5883 mabs × sec (4710–6955 mabs × sec)}, and it was significantly higher in the patients with ACI than in the HVs and patients with CLD (Figure 4B). Regarding the AUC value in the ROC analyses of ACI or AMI vs. non-thrombosis, the AUC of CWA-APTT and CWA-sTF/FIXa was >0.800 in AMI vs. HVs or CLD (Table 3A).

The reference interval of the AUC/second DPT of CWA-APTT and CWA-sTF/FIXa was 70.8–231 mabs and 37.2–118 mabs, respectively. The AUC/second DPT of CWA-APTT was significantly higher in the patients with cancer {212 mabs (165–278 mabs)}, AMI {265 mabs (186–342 mabs)}, and ACI {212 mabs (156–258 mabs)} than in the HVs {135 mabs (105–163 mabs)} and CLDs {165 mabs (133–210 mabs)}, and it was significantly higher in the patients with AMIs than in the HVs, the patients with cancers, and the patients with CLD (Figure 4C). The AUC/second DPT of CWA-sTF/FIXa was significantly higher in the patients with AMI {128 mabs (103–176 mabs)} than in the HVs {64.4 mabs (57.0–75.9 mabs)} and the patients with ACI {107 mabs (80.9–133 mabs)}, cancer {95.2 mabs (77.0–128 mabs)}, and CLD {86.3 mabs (72.0–100 mabs)}, and it was significantly higher in the patients with ACI than in the HVs and the patients with CLD (Figure 4D). Regarding the AUC value from the ROC analyses of the CWA-AUC/second DPT for ACI or AMI vs. non-thrombosis, the CWA-APTT showed that it was >0.800 in patients with AMI or ACI vs. HVs, and the CWA-sTF/FIXa showed that it was >0.900 in patients with AMI or ACI vs. HVs (Table 3B).

## 4. Discussion

Routine measurements of clotting time, such as APTT and PT, are used to evaluate hemostatic abnormality, clotting factor deficiencies [32], inhibitors of clotting factor [33], DIC [34], and lupus anticoagulant (LA) [35] and to monitor anticoagulants such as heparin or warfarin [36,37,38]; however, these measurements are not able to detect a hypercoagulable state as reliably as a thrombin generation test [39] or thromboelastography [40]. Our findings show no significant difference in the peak times of CWA-APTT between patients with and without thrombosis. However, the evaluation of the APTT or PT has been updated since the development of the CWA, including the modified CWA [30,31]. The second peak time of CWA-sTF/FIXa was significantly shorter in the patients with ACI than in the HVs. In addition, a CWA shows not only the peak time but also the peak height, peak width, and AUC [30,31]. The detection of a hypercoagulable state using the elevated peak height of CWA-APTT and CWA-sTF/FIXa has been reported in patients with malignant neoplasm [41] and ACI [21]. Our findings also show that the patients with AMI were in a hypercoagulable state, as were the patients with cancer or ACI. In the thrombin generation test [42,43], a shortened peak time and increased AUC indicate a hypercoagulable state. Therefore, the AUC and AUC/second DPT of CWA-APTT or CWA-sTF/FIXa may indicate a hypercoagulable state or thrombogenicity, respectively. A ROC analysis of the patients with AMI or ACI vs. the patients with non-thrombotic diseases showed that the usefulness in detecting a hypercoagulable state was in the order of AUC/second DPT, AUC, and peak height; CWA-sTF/FIXa was better than CWA-APTT. As these AMI patients with hypercoagulability by CWA had low D-dimer levels, CWA-APTT and CWA-sTF/FIXa can be independent markers for hypercoagulability from fibrin-related markers.

Patients with AMI or atherosclerotic ACI are generally treated with antiplatelet therapy [44,45], and hypercoagulability has not been considered important in these patients. Our CWA-APTT and CWA-sTF/FIXa results showed that the patients with ACI or AMI were markedly hypercoagulable. Indeed, atherosclerotic ACI patients are treated with antithrombin agents such as argatroban [46]. Many relationships between the hypercoagulable state and AMI have also been reported [47,48,49]. During atherosclerotic plaque rupture, the hypercoagulable state may increase the size of the coronary artery thrombosis, causing severe AMI. However, a hypercoagulable state might not be observed in patients with AMI induced by coronary spasm [50]. It has been reported that oral anticoagulants with or without aspirin do not reduce mortality, reinfarction, or stroke but significantly increase major bleeding [51]. However, when percutaneous coronary intervention is performed in patients with AMI, they are usually treated with unfractionated heparin and antiplatelets [52]. Therefore, the peak times of CWA-APTT and CWA-sTF/FIXa were slightly prolonged in the AMI patients in our study. In these patients, the effect of heparin was confirmed based on thrombin time and an anti-Xa assay. Patients with lupus anticoagulant were excluded from this study. sTF/FIXa using PRP can be used to evaluate the effect of platelets. AMI is considered to have major effects via platelet activation. There was no significant difference in platelet count between the patients with AMI and CLD. There was a significant difference in sTF/FIXa between the patients with and without thrombocytopenia (platelet count < 8 × 10^9^/L) [29,30,31]. This thrombocytopenia, which was observed in liver cirrhosis, was excluded from CLD in this study.

The mechanism underlying the development of a hypercoagulable state in AMI is considered to involve various underlying diseases, such as diabetes mellitus [53], hypertension [54], hyperlipidemia [55], inflammation [56], malignant neoplasm [57], and obesity [58]. These risk factors cause mild or strong hypercoagulable states owing to the increased expression of TF [59], the activation of the coagulation system [60], inflammatory cytokines [61], atherosclerosis [55], platelet activation [62], and thrombin burst [63]. These factors increase the peak heights of CWA-APTT and CWA-sTF/FIXa and shorten the peak time of CWA-sTF/FIXa [31,64]. “The peak height × peak width” half of the velocity curve is similar to the AUC of the velocity curve. Although there was no significant difference in the peak widths of CWA-APTT and CWA-sTF/FIXa between the patients with and without thrombosis, the AUC and AUC/peak time of the acceleration curve were strongly reflected in the hypercoagulable state.

In terms of clinical implication, this study demonstrates hypercoagulability in patients with AMI or ACI by using CWA-APTT or CWA-sTF/FIXa, thus suggesting that anticoagulant therapy may be useful for AMI or ACI. Evidence of hypercoagulability in AMI or ACI indicates a thrombotic risk for these diseases, suggesting the possibility of the prophylaxis of AMI or ACI.

### Limitation of Reference Interval of CWA

APTT (the second DPT of CWA-APTT) has sufficiently been established; however, the establishment of a reference interval for other parameters of CWA-APTT and CWA-sTF/FIXa is proceeding. APTT is automatically obtained from a fully automatic blood coagulometer, whereas the other parameters of CWA-APTT and all parameters of CWA-sTF/FIXa are manually measured in each patient, suggesting that large-scale study using CWA may be slightly difficult. In addition, there are a few HVs who have normal coagulation ability at old age. Therefore, it is difficult to use age-matched controls in a CWA study.

## 5. Conclusions

An increased peak height and AUC of CWA-sTF/FIXa and a shortened second DPT of CWA-sTF/FIXa were observed in patients with AMI and ACI. The CWA-AUC/second DPT of CWA-sTF/FIXa may be useful for detecting a hypercoagulable state in AMI or ACI.

## Figures and Tables

**Table 1:** CWA-APTT and CWA-sTF/FIXa in HVs and patients with CLD, cancer, ACI, and AMI.

	Second Derivative		First Derivative			Fibrin Formation	
APTT	Peak Time	Peak Height	Peak Time	Peak Width	Peak Height	Peak Time	Peak Height
HVs	36.8 (35.6–38.9)	731 (600–804)	34.7 (33.7–36.6)	36.0 (31.1–42.0)	231 (201–273)	36.8 (35.6–38.9)	196 (171–214)
CLD	35.8 (33.1–38.4)	888 *** (752–1033)	33.8 (31.4–36.5)	35.0 (31.5–42.1)	292 *** (246–339)	35.8 (33.1–38.4)	220 *** (198–250)
Cancer	36.8 (34.1–38.2)	955 *** (824–1168)	34.3 (31.8–36.1)	41.1 *** (34.9–46.5)	315 *** (268–394)	36.8 (34.1–38.2)	253 *** (217–308)
ACI	35.8 (33.3–38.1)	997 *** (853–1191)	33.7 (31.4–36.1)	37.7 (33.0–42.8)	327 *** (276–404)	35.8 (33.3–38.1)	239 *** (211–282)
AMI	39.2 ** (35.4–44.8)	1069 *** (865–1374)	37.6 ** (33.6–42.0)	42.5 *** (37.4–50.5)	400 *** (324–495)	39.2 ** (35.4–44.8)	324 *** (273–395)
sTF/FIXa	Peak time	Peak height	Peak time	Peak width	Peak height	Peak time	Peak height
HVs	71.3 (65.1–79.2)	31.9 (28.7–40.0)	91.3 (85.8–98.0)	145 (134–158)	65.0 (55.4–71.9)	92.2 (86.9–97.8)	315 (255–373)
CLD	68.2 * (61.5–64.7)	44.5 *** (36.6–56.9)	84.5 *** (78.8–90.4)	138 *** (126–145)	83.6 *** (71.3–101)	85.7 *** (79.7–91.3)	330 (277–380)
Cancer	70.5 (62.7–79.3)	43.1 *** (31.3–62.1)	90.1 (79.8–100)	152 (131–170)	88.3 *** (73.2–114)	92.4 (81.7–102)	344 * (284–432)
ACI	63.8 ** (54.3–74.0)	47.0 *** (36.2–60.1)	85.3 ** (77.0–94.4)	142 (132–154)	91.1 *** (75.6–118)	85.8 *** (78.2–94.6)	347 ** (302–413)
AMI	68.1 (56.6–77.4)	46.8 *** (33.0–60.2)	93.0 (84.0–107)	162 *** (142–183)	112 *** (81.1–138)	94.6 (84.5–108)	473 *** (393–550)

CWA, clot waveform analysis; APTT, activated partial thromboplastin time; sTF/FIXa, small amount of tissue factor activation assay; HVs, healthy volunteers; CLD, chronic liver disease, ACI, acute cerebral infarction; AMI, acute myocardial infarction; *, *p* < 0.05; **, *p* < 0.01; ***, *p* < 0.001 in comparison to HVs.

**Table 2:** Area under the curve of ROC in CWA-APTT and CWA-sTF/FIXa (AMI or ACI vs. without thrombosis).

		ACI vs.	HVs	CLD	Cancer	AMI vs.	HVs	CLD	Cancer
CWA- APTT	Second DPT		0.658	0.537	0.538		0.609	0.652	0.675
	First DPT		0.620	0.523	0.523		0.660	0.677	0.710
	First DPW		0.562	0.573	0.605		0.741	0.751	0.584
	FFT		0.623	0.509	0.547		0.650	0.673	0.689
	Second DPH		0.859	0.637	0.519		0.810	0.655	0.565
	First DPH		0.857	0.646	0.530		0.901	0.774	0.681
	FFH		0.809	0.625	0.558		0.963	0.876	0.738
CWA- sTF/FIX	Second DPT		0.658	0.573	0.619		0.581	0.501	0.546
	First DPT		0.649	0.513	0.599		0.543	0.694	0.568
	First DPW		0.553	0.597	0.577		0.681	0.795	0.619
	FFT		0.661	0.509	0.547		0.548	0.673	0.689
	Second DPH		0.747	0.538	0.551		0.704	0.510	0.517
	First DPH		0.867	0.594	0.537		0.870	0.699	0.643
	FFH		0.636	0.589	0.515		0.844	0.826	0.750

CWA, clot waveform analysis; APTT, activated partial thromboplastin time; sTF/FIXa, small amount of tissue factor activation assay; HVs, healthy volunteers; CLD, chronic liver disease, ACI, acute cerebral infarction; AMI, acute myocardial infarction; DPT, derivative peak time; DPW, derivative peak width; FFT, fibrin formation time; DPH, derivative peak height; FFH, fibrin formation height; a red number indicates area under the curve > 0.800.

**Table 3:** ROC analysis of the AUC (A) and AUC/second DPT (B) of CWA-APTT and CWA-sTF/FIXa (AMI or ACI vs. without thrombosis).

(A) AUC		Cutoff	Sensitivity	Odds Ratio
CWA-APTT				
AMI vs. HVs	0.934	5362	85.6%	31.2
AMI vs. CLD	0.821	6526	74.3%	8.68
AMI vs. Cancer	0.679	7281	63.6%	3.03
ACI vs. HVs	0.812	4392	74.0%	4.82
ACI vs. CLD	0.648	5801	62.8%	2.93
ACI vs. Cancer	0.534	6479	51.2%	1.05
CWA-sTF/FIXa				
AMI vs. HVs	0.929	5774	87.4%	42.5
AMI vs. CLD	0.816	6890	74.3%	7.25
AMI vs. Cancer	0.696	7712	67.0%	4.18
ACI vs. HVs	0.833	5336	74.0%	8.10
ACI vs. CLD	0.635	6157	58.5%	2.01
ACI vs. Cancer	0.506	6587	51.6%	1.16
**(B) AUC/Second DPT**		**Cutoff**	**Sensitivity**	**Odds Ratio**
CWA-APTT				
AMI vs. HVs	0.867	169	78.8%	13.2
AMI vs. CLD	0.783	106	74.3%	8.57
AMI vs. Cancer	0.603	222	55.8%	1.12
ACI vs. HVs	0.836	158	74.0%	8.09
ACI vs. CLD	0.665	188	62.0%	2.66
ACI vs. Cancer	0.526	212	49.6%	1.00
CWA-sTF/FIXa				
AMI vs. HVs	0.912	79.8	85.4%	36.0
AMI vs. CLD	0.771	107	73.8%	7.86
AMI vs. Cancer	0.691	116	65.4%	3.62
ACI vs. HVs	0.867	76.5	78.0%	13.2
ACI vs. CLD	0.662	94.5	62.2%	2.75
ACI vs. Cancer	0.553	102	57.2%	1.83

AUC, area under the curve; CWA, clot waveform analysis; APTT, activated partial thromboplastin time; sTF/FIXa, small amount of tissue factor activation assay; HVs, healthy volunteers; CLD, chronic liver disease; ACI, acute cerebral infarction; AMI, acute myocardial infarctions; DPT, derivative peak time; a red number indicates area under the curve > 0.800.

## Data Availability

The data presented in this study are available on request from the corresponding author. The data are not publicly available due to privacy restrictions.
